# Natural Language Processing-Assisted Incidental Pulmonary Nodule Evaluation Program: Impact on Lung Cancer Outcomes

**DOI:** 10.3390/medsci14010104

**Published:** 2026-02-21

**Authors:** Noa Tamam Shenholz, Keren Hod, Liat Toderis, Noam Fink, Arnon Makori, Michael Peer, Evgeni Gershman, Merav A. Ben-David, Elizabeth Dudnik

**Affiliations:** 1 Faculty of Health Sciences, Joyce & Irving Goldman Medical School, Ben Gurion University of the Negev, Beer-Sheva 8410501, Israel; noatamam95@gmail.com (N.T.S.); meravak@assuta.co.il (M.A.B.-D.); 2 Department of Nutritional Sciences, School of Health Sciences, Ariel University, Ariel 4070000, Israel; hodkeren@gmail.com; 3 Research Department, Assuta Medical Centers, Tel-Aviv 6971028, Israel; 4 Department of Information Technology, Assuta Medical Centers, Tel-Aviv 6971028, Israel; liattod@gmail.com; 5 Industrial Engineering & Management Department, Ariel University, Ariel 4070000, Israel; 6 Assuta Medical Centers, Tel-Aviv 6971028, Israel; noamfi@assuta.co.il; 7 Faculty of Medicine, Hebrew University of Jerusalem, Jerusalem 9112102, Israel; 8 Imaging Services, Assuta Medical Centers, Tel-Aviv 6971028, Israel; arnonma@assuta.co.il; 9 Department of Thoracic Surgery, Tel Aviv Medical Center, Tel-Aviv 6423906, Israel; peer_mi@mac.org.il; 10 Faculty of Medicine, Tel-Aviv University, Tel-Aviv 6997801, Israel; drgershmane@gmail.com; 11 Pulmonary Institute, Tel Aviv Medical Center, Tel-Aviv 6423906, Israel; 12 Department of Oncology, Assuta Medical Centers, Tel-Aviv 6971028, Israel; 13 Thoracic Oncology, Assuta Medical Centers, Tel-Aviv 6971028, Israel

**Keywords:** lung cancer, NLP, natural language processing, IPN, incidental pulmonary nodule

## Abstract

**Introduction**: Early detection and timely treatment (Tx) initiation are critical to improving lung cancer (LC) outcomes. This study assessed the natural language processing (NLP)-assisted incidental pulmonary nodule (IPN) evaluation program, which employs chest computer tomography (CT) report analysis as an LC diagnostic screening (LCS) tool to identify suspicious lung findings (SLF) necessitating further investigation, and evaluated its impact on prognosis and diagnostic work-up and Tx timelines for patients with LC. **Materials and Methods**: Consecutive LC patients (n = 200) diagnosed at Assuta Medical Centers (AMC) between January 2019 and December 2022 were retrieved from the AMC electronic database using the MDClone big data platform, and divided into two groups: group A (NLP-assisted IPN evaluation, n = 100) and group B (traditional referral for evaluation of SLF by the community physician, n = 100). Stage at diagnosis, different diagnostic work-up and Tx timelines, and overall survival (OS) were assessed. **Results**: The NLP-assisted IPN evaluation program led to a significant stage shift (stage I disease: 48% vs. 27% in groups A and B, respectively, *p* = 0.013). Although the time from imaging to Tx initiation was similar (2.1 ± 5.3 months vs. 2.6 ± 5.9 months in groups A and B, respectively, *p* = 0.654), the time to systemic Tx (*p* = 0.035) and the time to radiotherapy (*p* = 0.044) were significantly shorter in group A. **Conclusions**: Implementing an NLP-assisted IPN evaluation program may enable earlier LC detection, driving a stage shift towards earlier diagnosis, improved diagnostic efficiency, and expedited time-critical interventions.

## 1. Introduction

Lung cancer (LC) remains the leading cause of cancer mortality worldwide (18.4% of the total cancer deaths), making early detection and timely treatment initiation critical to improving outcomes [1,2,3]. Delays along the diagnostic pathway are common and are clinically significant since time-to-treatment correlates with survival. Therefore, reducing the time from the initial presentation to the definitive treatment delivery in such an aggressive disease is critical [4,5]. Health-system data demonstrated that, once suspicious lung findings (SLF) are present, the imaging-to-treatment timetable is typically 2–3 months [6]. Although low-dose chest computer tomography (LDCT) screening programs facilitate early LC diagnosis, they have significant downsides. Specifically, current age/pack-year eligibility fails to cover many at-risk individuals leaving a sizeable gap in early detection [7,8,9,10,11].

To address issues related to rapid LC diagnosis and in the absence of country lung cancer screening (LCS) programs, in January 2022, Assuta Medical Centers (AMC) established a natural language processing (NLP)-assisted incidental pulmonary nodule (IPN) evaluation program to surface IPNs from chest computer tomography (CT) reports, trigger clinician review, and coordinate expedited work-ups. Modern IPN programs offer a complementary, “opportunistic screening” pathway by capturing nodules discovered on routine clinical CTs and moving them through structured workflows: automated case identification, registry/longitudinal tracking, guideline-based management, and rapid escalation to multidisciplinary review [12,13,14,15]. Scaling this model requires automating high-volume text processing. NLP can mine free-text radiology reports to surface IPNs in near-real time, trigger clinician review, and reduce tracking failures, addressing both throughput and coordination while easing radiologist workload [16,17,18]. Whether the introduction of IPN programs results in a stage shift and affects prognosis of LC is of interest, and large studies are needed to establish their impact.

Within this context, we conducted a retrospective cohort analysis focusing on LC patients and comparing an NLP-assisted IPN evaluation pathway with a standard referral pathway (traditional SLF referral for evaluation by the community physician), testing whether the novel approach shifts LC stage at diagnosis and shortens key time intervals from first cancer-related imaging to diagnosis and treatment.

## 2. Materials and Methods

### 2.1. Study Design

This study was designed as a retrospective comparative cohort analysis aimed at evaluating the impact of implementing NLP-assisted IPN evaluation programs on the disease stage, different diagnostic work-up and treatment timelines, and overall survival (OS) for LC patients.

The study compared outcomes for LC patients referred for diagnostic evaluation using the traditional referral method (patients with SLF diagnosed and referred for evaluation by community physicians) versus those evaluated with the newly implemented NLP-assisted IPN evaluation protocol (the use of NLP-based algorithms to extract the data from the CT reports and reveal the patients in whom further clinical evaluation of IPN is required, which was introduced in AMC in January 2022).

At AMC, development and internal assessment of the NLP-assisted algorithm comprised: (1) retrospective review of chest CT reports from lung cancer patients; (2) definition of keyword logic for NLP algorithm implementation, requiring co-occurrence of two term categories—phrases indicating IPN suspicion and phrases recommending further evaluation/work-up; and (3) deployment of the algorithm on a larger retrospective database to assess performance, prioritizing sensitivity over specificity given the prespecified manual image review step. Of note, this was an internal, pragmatic assessment and no formal external validation was performed. NLP-based algorithms employed MDClone NLP Studio platform (Beer Sheba, Israel) which uses a deterministic pattern-recognition engine built on regular expression logic. The platform’s core component, the MDClone-R interpreter, identifies recurring textual patterns and character sequences without reliance on linguistic syntax or semantic modeling, thus enabling application across multiple languages and documentation styles. Further evaluation of NLP-identified IPNs included manual image review by a thoracic oncologist, followed by discussion at a weekly multidisciplinary thoracic tumor board to guide the diagnostic work-up and management (the board is composed of a thoracic radiologist, thoracic oncologists, pulmonology specialists, thoracic surgeons, and radiation oncologists, and is supported by a nurse who coordinates communication with patients and referring physicians).

Patients were divided into two main groups: group A (NLP-assisted IPN evaluation protocol), and group B (standard of care [SOC], the traditional referral method). The co-primary study objectives were to assess the impact of NLP-assisted IPN evaluation program on disease stage at diagnosis, and whether this approach reduced the time from the first imaging study indicating cancer-related findings to the initiation of oncological treatment. Secondary study objectives included comparing the time from the first CT-based imaging study (either chest CT, total body CT, cardiac CT, or spine CT) indicating cancer-related lung findings to biopsy, the time to establishing histological diagnosis, and the time to molecular tumor subtype determination. Furthermore, the time from the first CT-based imaging study indicating cancer-related findings to oncological treatment was assessed separately for the three main oncological treatment types (surgery, radiotherapy, and systemic therapy). OS was also analyzed.

### 2.2. Data Collection

Patient data was collected retrospectively from the electronic medical records (EMRs) and encoded into the excel spreadsheet for the analysis. Data was collected using the MDClone big data platform, which integrates data from diverse sources and components of the EMR within AMC to provide streamlined access to structured and unstructured patient information and enables retrieval of comprehensive patient data. The data accuracy and completeness were further manually re-checked.

The study included 200 consecutive patients diagnosed with LC from January 2019 to December 2022. Only patients with histologically confirmed lung cancer who received treatment and follow-up at AMC during the study period were included, regardless of whether the initial CT-based imaging was prompted by symptoms or revealed incidental findings requiring further evaluation. Patients were excluded if the key data were missing (such as the date of the initial CT-based imaging study), if they received treatment at another facility, or if they were lost to follow-up after the diagnosis. This detailed selection process ensured that the final cohort was representative of the LC population treated at AMC during the specified timeframe.

Data collection focused on identifying relevant clinical timelines and important prognostic characteristics. Prognostic characteristics included demographic information, smoking history, pathological and molecular characteristics, weight loss, and Eastern Cooperative Oncology Group performance status (ECOG PS). Key patient medical journey dates were extracted, including the date of the first CT-based imaging study demonstrating cancer-related findings, date of biopsy, date of obtaining histological diagnosis, and date of molecular subtype determination. Additionally, type and date of different oncological treatments (surgery, radiotherapy, or systemic therapy) were recorded. Outcome information, such as death status, date of death, and date of last follow-up, were documented.

### 2.3. Statistical Analysis

Baseline and treatment characteristics, treatment timelines and OS were compared between the groups.

Data was analyzed using ChatGPT Plus, version 4.0, and analyses were conducted using Python, version 3.11.2. Categorical variables were summarized as counts and percentages and compared using Pearson’s Chi-squared test. For categorical variables with expected frequencies below 5 in any cell, Fisher’s exact test was applied. Continuous variables (e.g., age and diagnostic time intervals) were reported as mean ± standard deviation (SD) and median with a 95% confidence interval (CI), as appropriate. Comparisons of continuous variables between groups were performed using the Mann–Whitney U test, due to the non-parametric nature of the data. OS was estimated using the Kaplan–Meier method. The median OS values for each group with corresponding 95% CIs, along with the OS probabilities with corresponding 95% CIs at 6 and 12 months were obtained from the Kaplan–Meier curves. Survival distributions were compared between groups using the log-rank (Mantel–Cox) test, and the two-sided *p*-value from this test was reported to assess statistical significance. Follow-up time was summarized using the reverse Kaplan–Meier method. A two-sided *p*-value < 0.05 was considered statistically significant.

### 2.4. Ethical Statement

The study was conducted in accordance with the ethical standards, ensuring full patient confidentiality. Approval from the Assuta Institutional Helsinki Committee was obtained prior to study initiation (protocol approval number ASMC-0005-22).

## 3. Results

### 3.1. Baseline and Treatment Characteristics

A total of 200 consecutive LC patients were included in the study: group A (NLP-assisted IPN evaluation protocol), n = 100; and group B (SOC, traditional referral method), n = 100.

Baseline and treatment patient characteristics are displayed in Table 1. The cohort consisted predominantly of older adults with a slight male predominance and high rates of smoking, reflecting typical demographics of LC populations. Importantly, only 59% of patients (58% and 60% of patients in groups A and B, respectively, *p* = 0.745), met the modern American Cancer Society (ACS) eligibility criteria for LCS with LDCT (age 50–80 years and current or past smoking ≥ 20 pack-years). Most patients had a good performance status (ECOG PS 0–1) and experienced no weight loss. Adenocarcinoma was the most common histological subtype, epidermal growth factor receptor gene (EGFR) mutations and Kirsten rat sarcoma virus (KRAS) mutations were the most frequently detected molecular alterations, while other driver mutations were rare—all the above-mentioned factors, along with the tumor programmed cell death ligand-1 (PD-L1) expression distribution, were typical for the non-small cell lung cancer (NSCLC) population.

**Figure 1:**
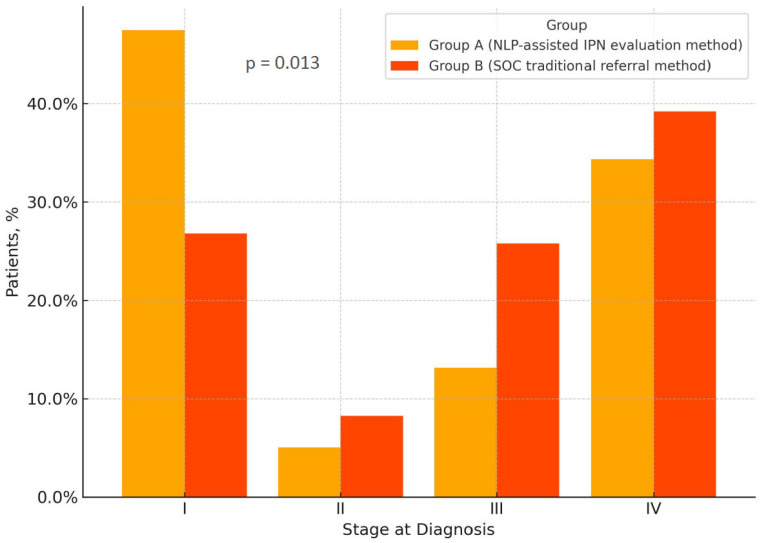
Stage distribution at diagnosis by study group for all lung cancer patients in the cohort (n = 200); group A (NLP-assisted IPN evaluation protocol, n = 100), group B (SOC, traditional referral method n = 100). Abbreviations: IPN—incidental pulmonary nodule, NLP—natural language processing, SOC—standard of care.

The demographic, baseline clinical, pathological and molecular characteristics were similar in both groups, with no significant differences in terms of age, sex, smoking history, ECOG PS, weight loss, tumor histology and molecular tumor characteristics (Table 1). More patients in group A were diagnosed at an earlier disease stage (stage shift) with a much higher proportion of patients in group A diagnosed at stage I, as compared to patients in group B (48% vs. 27%, respectively, *p* = 0.013; Table 1, Figure 1).

The main differences in treatment approaches between the groups included higher use of chemo-immunotherapy, higher rates of stereotactic body radiation therapy (SBRT) and lower rates of definitive conformal radiotherapy and palliative radiotherapy in group A as compared to group B, reflecting different treatment approaches in early-stage and advanced-stage NSCLC and broader implementation of neo-adjuvant chemo-immunotherapy in the treatment of NSCLC (Table 1).

### 3.2. Diagnostic and Treatment Timelines

**Figure 2:**
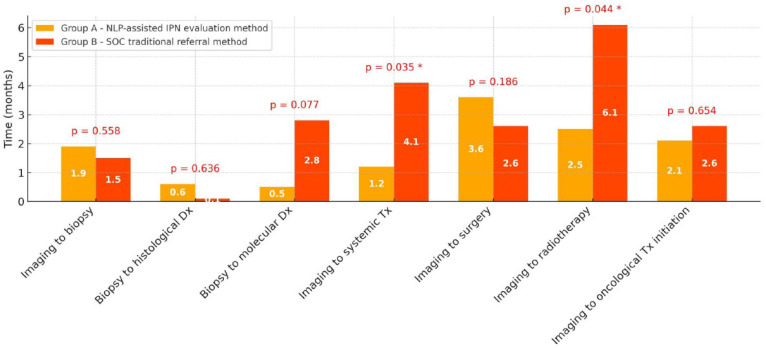
Diagnostic and treatment timelines by study group for all lung cancer patients in the cohort (n = 200), presented by mean values, months; group A (NLP-assisted IPN evaluation protocol, n = 100), group B (SOC, traditional referral method n = 100). Abbreviations: Dx—diagnosis, IPN—incidental pulmonary nodule, NLP–natural language processing, SOC—standard of care, Tx—treatment. * indicates a statistically significant difference.

Across the cohort, timelines from the initial imaging study demonstrating any cancer-related finding to subsequent diagnostic and therapeutic steps varied between groups. Diagnostic and treatment timeline comparisons are presented using both mean values (Figure 2) and median values (Figure S1).

Regarding the interval from the first CT-based imaging demonstrating any cancer-related finding to biopsy, the mean time was similar between groups (1.9 ± 4.7 months in group A vs. 1.5 ± 5.2 months in group B, *p* = 0.558), with no statistically significant difference also observed in the median values. Similarly, the interval from biopsy to histological diagnosis did not differ significantly (0.6 ± 4.0 months vs. 0.1 ± 6.1 months, *p* = 0.636). The time from biopsy to molecular diagnosis showed a numerically shorter mean value in group A (0.5 ± 2.9 months) compared to group B (2.8 ± 5.3 months), approaching statistical significance (*p* = 0.077), suggesting a trend toward faster molecular work-up with the NLP-assisted IPN evaluation protocol.

No statistically significant differences between groups were found in the intervals from first imaging demonstrating any cancer-related finding to initiation of any oncological treatment (2.1 ± 5.3 months in group A vs. 2.6 ± 5.9 months in group B, *p* = 0.654). However, a statistically significant difference was observed in the interval from first imaging demonstrating any cancer-related finding to first-line systemic therapy (1.2 ± 5.3 months in group A vs. 4.1 ± 5.8 months in group B, *p* = 0.035), indicating more rapid treatment initiation with the NLP-assisted IPN evaluation pathway. Similarly, time from first imaging demonstrating any cancer-related finding to radiotherapy was significantly shorter in group A (2.5 ± 5.5 months) compared to group B (6.1 ± 7.6 months, *p* = 0.044). No statistically significant differences were found in the intervals from imaging to surgery; however, timelines numerically favored group B (3.6 ± 4.7 months in group A vs. 2.6 ± 6.9 months in group B, *p* = 0.186).

### 3.3. Overall Survival Analysis

**Figure 3:**
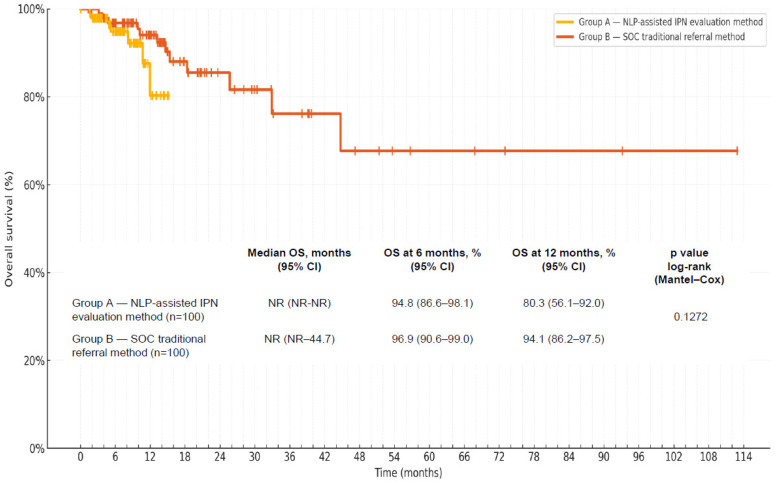
Overall survival for all lung cancer patients in the cohort (n = 200): group A (NLP-assisted IPN evaluation protocol, n = 100), group B (SOC, traditional referral method n = 100). Abbreviations: CI(s)—confidence interval(s), IPN—incidental pulmonary nodule, NLP—natural language processing, NR—not reached, OS—overall survival, SOC—standard of care.

The median follow-up period was short; it comprised 6.8 months and 14.5 months in groups A and B, respectively. At the time of last follow-up, 7 and 12 deaths were observed in groups A and B, respectively. Kaplan–Meier OS analysis could not identify a statistically significant difference between groups over the available follow-up (log-rank *p* = 0.1272; Figure 3). Median OS was not reached in either group and point OS estimates at 6 and 12 months had overlapping 95% CIs, indicating substantial uncertainty regarding the comparison between the groups (Figure 3).

## 4. Discussion

The results of this comparative retrospective cohort study emphasized the importance of the IPN diagnostic approach as an LCS tool and demonstrated the value of NLP implementation in facilitating early LC detection and rapid diagnostic evaluation.

For instance, NLP-assisted IPN evaluation program implementation demonstrated an important stage shift towards earlier stages at diagnosis, whereas no other differences in major demographic characteristics between the SOC evaluation group and the NLP-assisted IPN evaluation group were seen. Specifically, a higher percentage of cases were diagnosed at Stage I in the NLP-assisted IPN evaluation cohort as opposed to the SOC evaluation cohort (48% vs. 27%, respectively, *p* = 0.013), highlighting the significance of the stage shift and emphasizing the potential impact of the NLP-assisted IPN diagnostic approach implementation on the early LC detection. NLP-assisted IPN evaluation also shortened the time to first-line systemic therapy initiation (*p* = 0.035) and the time to radiotherapy initiation (*p* = 0.044). Unfortunately, the OS impact of NLP-assisted IPN evaluation program implementation in the analyzed cohort could not be accurately estimated, considering the short follow-up and low event counts, limiting the power and precision and weakening the validity of between-group comparisons over time.

Importantly, multiple reports have demonstrated that structured IPN programs have the potential to shift stage at diagnosis towards early-stage disease and augment early LC detection in routine practice beyond the use of LDCT screening. Most of the reports, however, are either non-comparative, or do not use the SOC evaluation cohort for the proper comparison. Specifically, the hospital system that deployed a patient-tracking system and computerized nodule registry reported a higher frequency of stage I NSCLC among IPN-detected cancers after as compared to before its implementation, emphasizing the impact of organized follow-up infrastructure [12]. In addition, real-world cohort data from another dedicated IPN follow-up program found substantial cancer yield with predominantly early-stage presentation: 6% of >4000 patients developed LC with ~77% potentially curable stage at diagnosis [13]. In a prospective, multi-facility cohort from the Mississippi Delta (22 facilities across a 125-county service area; 7121 LCS participants and 22,455 IPN participants), Liao and colleagues reported three-year cumulative lung cancer diagnosis rates of 4.9% in the LCS cohort using the US Preventive Services Task Force (USPSTF) 2021 LCS eligibility criteria and 4.3% in the IPN cohort, emphasizing the importance of augmentation of LCS by the implementation of the IPN program [7]. Overall, multiple reviews and single-center IPN-clinic experiences similarly note very high proportions of stage I–II disease among incidentally detected cancers when nodules are managed through standardized clinics [19]. Modeling work further suggests that earlier recognition of incidental nodules could translate into measurable mortality reductions, although the magnitude depends on growth kinetics and timelines of action [20]. Conceptually, expert commentary in thoracic oncology frames IPN pathways as an opportunity to complement screening, expanding the reach of early detection beyond the small fraction of eligible, screened individuals [21].

Taken together, these studies align with our observed stage shift under the NLP-assisted IPN evaluation program and support IPN management as a practical lever to broaden early lung cancer detection, while reinforcing the need for organized pathways, adherence monitoring, and longer follow-up to confirm survival benefits.

Of note, only 59% of patients in the analyzed cohort met the modern ACS eligibility criteria for LCS with LDCT (age 50–80 years and current or past smoking ≥20 pack-years), underscoring a broader problem: current age/pack-year rules underestimate the population impact of LCS. The eligibility rates for LCS in the IPN cohort using the USPSTF 2021 criteria as low as 13% were reported by Liao and colleagues [7]. Multivariable, risk-prediction approaches (e.g., Prostate, Lung, Colorectal, and Ovarian 2012 model [PLCOm2012]) consistently identify more cancers at screening with better efficiency than categorical thresholds, missing far fewer cancers and improving sensitivity without sacrificing specificity; comparative modeling likewise shows that risk-based strategies avert more deaths and yield more life-years than USPSTF-style criteria [8,9]. In parallel, emerging evidence from Asia indicates that targeted LDCT in carefully selected populations of never-smokers can detect substantial numbers of cancers, predominantly early stage, supporting a role for multifactorial criteria that go beyond smoking history alone; cost-effectiveness appears context-dependent but can be favorable in some settings [10,11]. Overall, these findings suggest that maximal population benefit from early LC detection will come from risk-based LCS plus an organized IPN program, rather than applying screening eligibility criteria defined only by age and smoking intensity.

Despite its promise, real-world LDCT implementation is constrained by radiologist workload which scales sharply when screening programs expand. Contemporary reviews emphasize that artificial intelligence (AI) used as a first reader/triage can reduce workload by filtering likely negative studies while preserving safety as a second reader to boost sensitivity [22]. Equally important for scale is automation of IPN workflows for extraction of IPNs from free-text CT reports and support tracking, thereby reducing loss to follow-up and enabling earlier diagnosis [14,15,16,17,18]. Consistent with this evidence, IPN AMC program’s feasibility has depended on end-to-end automation (including NLP-driven report mining and registry functions), which addressed the same throughput and coordination challenges documented in the literature and made large-scale deployment practical. Notably, our study is the first to describe the deployment of a Hebrew-based NLP algorithm within an IPN program.

Furthermore, our study delved into the various diagnostic and treatment timelines of LC patients in the reported cohort and mainly focused on the time from first CT-based imaging demonstrating any cancer-related finding to initiation of any oncological treatment (2.1 ± 5.3 months and 2.6 ± 5.9 months, in the NLP-assisted IPN evaluation and SOC evaluation groups, respectively, *p* = 0.654). The obtained data aligns with a recent population-based analysis mapping the NSCLC patient journey, which reported on a 2.6 month timeline from imaging to primary treatment initiation [6].

Although the NLP-assisted IPN evaluation program used in the reported cohort did not demonstrate a statistically significant difference in the time from first imaging to initiation of any oncological treatment, it was indeed associated with significantly shorter times to certain treatment milestones, particularly systemic therapy and radiotherapy initiation, while maintaining comparable timelines for other diagnostic steps. These findings suggest that NLP-assisted IPN evaluation pathways may accelerate specific phases of the diagnostic-to-treatment process, potentially improving time-sensitive cancer care delivery.

Our study has several key limitations that temper the robustness of the conclusions. The retrospective, single-center, non-randomized pre/post design is inherently vulnerable to confounding and secular trends, including evolving systemic therapies and diagnostic practices, COVID-19-related fluctuations in service delivery, and selection bias (restricted to patients diagnosed, treated, and followed at AMC). Another key limitation is the lack of formal hand-read validation of the NLP algorithm, precluding robust report-level sensitivity/specificity estimates and systematic quantification of false-positive/false-negative rates. The markedly shorter follow-up in one cohort limits the interpretability of survival endpoints and in addition to the overall limited follow-up, few events unreached median OS and wide CIs. To the best of our knowledge, there were no internal organizational changes or technological renovations in care routine at AMC during 2019–2022 beyond implementation of the NLP-assisted IPN evaluation program; nonetheless, residual confounding from external time-related factors cannot be excluded. In addition, multiple diagnostic and treatment timeline endpoints were assessed without adjustment for multiplicity, increasing the risk of type I error. Generalizability may also be limited, as the pathway depends on Hebrew-based EMR/NLP infrastructure and a specific organizational model that may require adaptation and local validation elsewhere. Taken together, these limitations warrant cautious interpretation and underscore the need for prospective, multicenter studies with larger cohorts and longer follow-up to determine whether NLP-assisted IPN programs independently improve early lung cancer detection, management, and clinical outcomes.

## 5. Conclusions

In conclusion, our study highlights the value of the IPN diagnostic approach as an LCS tool and the transformative potential of integrating innovative technologies such as NLP into clinical practice to optimize LC care. Given the study design and limitations, prospective studies are needed to validate the observed associations and clarify any impact on clinical outcomes.

## Figures and Tables

**Table 1:** Baseline and treatment patient characteristics.

	Group A (NLP-Assisted IPN Evaluation Method, n = 100)	Group B (SOC, Traditional Referral Method, n = 100)	*p*-Value
Age, years			0.619
Mean ± SD	70.08 ± 10.05	69.42 ± 8.64	
Median, 95% CI	72 (32–99)	70 (37–87)	
Sex, %			0.777
Female	45	48	
Male	55	52	
Smoking history, %			0.868
Current/past smoker	77	75	
Never smoker	23	25	
Smoking, p/y			0.924
Mean ± SD	45.62 ± 27.98	46.09 ± 32.49	
Median, 95% CI	50.0 (1.0–150.0)	40.0 (3.0–150.0)	
ACS criteria (age 50–80 and ≥20 p/y), %			0.745
Meeting the criteria	58	60	
Not meeting the criteria	19	15	
NA	23	25	
Histological subtype, %			0.460
Adenocarcinoma	71	66	
Squamous cell carcinoma	15	17	
Small cell carcinoma	3	7	
NSCLC NOS	7	6	
Other	0	2	
Unknown	4	2	
Stage, %			0.013
I	48	27	
II	5	8	
III	13	26	
IV	34	39	
ECOG PS at diagnosis, %			0.299
0/1	89	93	
2/3/4	8	7	
NA	3	0	
Weight loss of more than 5%, %			0.764
Yes	24	21	
No	76	79	
PD-L1 TPS, %			0.709
≥50%	31	30	
1–49%	17	12	
0%	52	58	
Targetable alteration, %			
EGFR mutation			0.670
Yes	13	16	
KRAS mutation			0.961
Yes	9	10	
BRAF mutation			0.182
Yes	2	7	
cMet ex14 skipping mutation			0.668
Yes	5	3	
ALK re-arrangement			1.000
Yes	1	2	
ROS1 re-arrangement			0.521
Yes	0	2	
RET re-arrangement			0.992
Yes	1	0	
1L systemic treatment, %			0.016
Chemotherapy	12	32	
Targeted therapy	10	17	
ICI	2	9	
Combination of chemotherapy and ICI	20	14	
Surgery type, %			0.526
VATS lobectomy	35	28	
VATS sublobar resection	7	5	
Radiotherapy type, %			0.181
Definitive conformal radiotherapy	7	17	
SBRT	20	15	
Palliative	12	18	
SRS	10	11	

Abbreviations: 1L systemic therapy—first-line systemic treatment, ACS—American Cancer Society, ALK—anaplastic lymphoma kinase gene, BRAF—V-raf murine sarcoma viral oncogene homolog B1, c-Met ex14 skipping—tyrosine-protein kinase Met exon 14 skipping, CI(s)—confidence interval(s), ECOG PS—Eastern Cooperative Oncology Group performance status, EGFR—epidermal growth factor receptor gene, ICI—immune check-point inhibitors, IPN—incidental pulmonary nodule, KRAS—Kirsten rat sarcoma virus, NA—not specified/not available, NLP—natural language processing, NOS—not otherwise specified, NSCLC—non-small cell lung cancer, p/y—pack/years, PD-L1—programmed cell death ligand-1, RET—proto-oncogene RET, ROS1—proto-oncogene tyrosine-protein kinase ROS1, SBRT—stereotactic body radiation therapy, SD—standard deviation, SOC—standard of care, SRS—stereotactic radiosurgery, TPS—tumor proportion score, VATS—video-assisted thoracoscopic surgery.

## Data Availability

The original contributions presented in this study are included in the article/Supplementary Material. Further inquiries can be directed to the corresponding author(s).

## Supplementary Materials

The following supporting information can be downloaded at: https://www.mdpi.com/article/10.3390/medsci14010104/s1, Figure S1: diagnostic and treatment timelines by study group for all lung cancer patients in the cohort (n = 200), presented by median values, months; group A (NLP-assisted IPN evaluation protocol, n = 100), group B (SOC, traditional referral method n = 100). Abbreviations: Dx—diagnosis, IPN—incidental pulmonary nodule, NLP—natural language processing, SOC—standard of care, Tx—treatment.

## Abbreviations

1L systemic treatment—first-line systemic treatment, ACS—American Cancer Society, AI—artificial intelligence, ALK—anaplastic lymphoma kinase gene, AMC—Assuta Medical Centers, BRAF—V-raf murine sarcoma viral oncogene homolog B1, c-Met ex14 skipping—tyrosine-protein kinase Met exon 14 skipping, CI(s)—confidence interval(s), CT—chest computer tomography, Dx—diagnosis, ECOG PS—Eastern Cooperative Oncology Group performance status, EGFR—epidermal growth factor receptor gene, EMR—electronic medical records, ICI—immune check-point inhibitors, IPN—incidental pulmonary nodule, KRAS—Kirsten rat sarcoma virus, LC—lung cancer, LCS—lung cancer screening, LDCT—low-dose chest computer tomography, (m)OS—(median) overall survival, NA—not specified/not available, NLP—natural language processing, NOS—not otherwise specified, NR—not reached, NSCLC—non-small cell lung cancer, p/y—pack/years, PD-L1—programmed cell death ligand-1, PLCOm2012—Prostate, Lung, Colorectal, and Ovarian 2012 model, RET—proto-oncogene RET, ROS1—proto-oncogene tyrosine-protein kinase ROS1, SBRT—stereotactic body radiation therapy, SD—standard deviation, SLF(s)—suspicious lung finding(s), SOC—standard of care, SRS—stereotactic radiosurgery, TPS—tumor proportion score, Tx—treatment, USPSTF—United States Preventive Services Task Force, VATS—video-assisted thoracoscopic surgery.
