# Supplementary figures and images for: Natural Language Processing-Assisted Incidental Pulmonary Nodule Evaluation Program: Impact on Lung Cancer Outcomes
Source: Med Sci (Basel). 2026 Feb 21;14(1):104. doi: 10.3390/medsci14010104 (PMC13027686; doi:10.3390/medsci14010104)

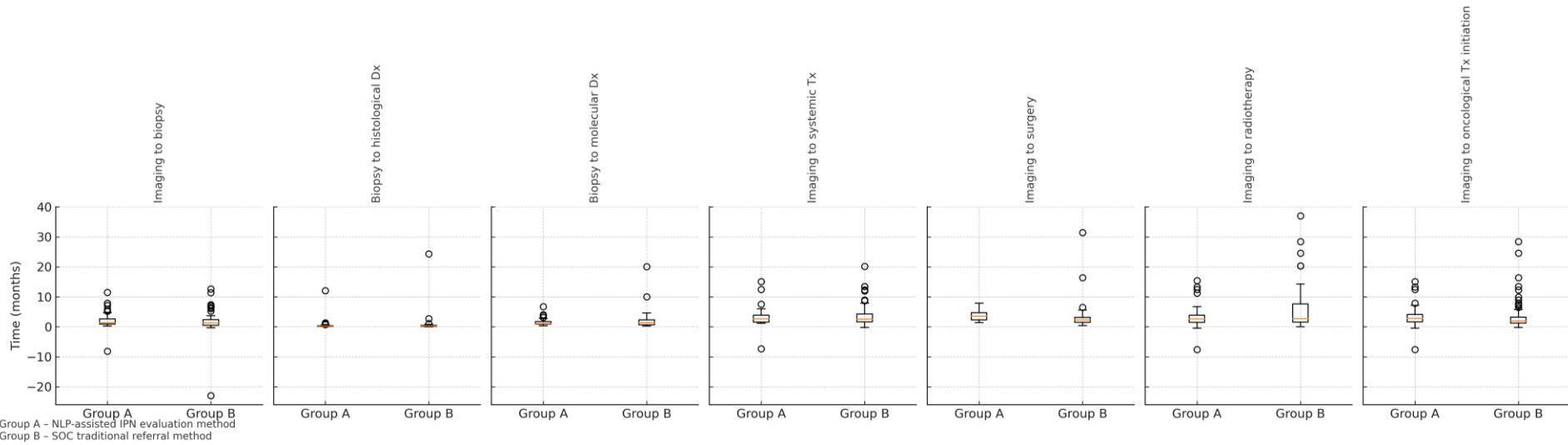

Supplement: Supplementary file 1 [file medsci-14-00104-s001.zip › medsci-4123835-Figure S1.pdf]
